# Vancomycin versus daptomycin for the treatment of methicillin-resistant *Staphylococcus aureus* bacteremia due to isolates with high vancomycin minimum inhibitory concentrations: study protocol for a phase IIB randomized controlled trial

**DOI:** 10.1186/1745-6215-15-233

**Published:** 2014-06-19

**Authors:** Shirin Kalimuddin, Rachel Phillips, Mihir Gandhi, Nurun Nisa de Souza, Jenny GH Low, Sophia Archuleta, David Lye, Thuan Tong Tan

**Affiliations:** 1 Department of Infectious Diseases, Singapore General Hospital, 20 College Road, Singapore 169856, Singapore; 2 Singapore Clinical Research Institute, 31 Biopolis Way, Singapore 138669, Singapore; 3 Centre of Quantitative Medicine, Duke-NUS Graduate Medical School, 8 College Road, Singapore 169857, Singapore; 4 Division of Infectious Diseases, University Medicine Cluster, National University Health System, Kent Ridge Road, Singapore 119228, Singapore; 5 Department of Medicine, Yong Loo Lin School of Medicine, National University of Singapore, Kent Ridge Road, Singapore 119228, Singapore; 6 Department of Infectious Diseases, Communicable Disease Centre, Tan Tock Seng Hospital, Moulmein Road, Singapore 308433, Singapore

**Keywords:** MRSA, Bacteremia, Daptomycin, Vancomycin

## Abstract

**Background:**

Vancomycin is the standard first-line treatment for methicillin-resistant S*taphylococcus aureus* bacteremia. However, recent consensus guidelines recommend that clinicians consider using alternative agents such as daptomycin when the vancomycin minimum inhibitory concentration is greater than 1 ug/ml. To date however, there have been no head-to-head randomized trials comparing the safety and efficacy of daptomycin and vancomycin in the treatment of such infections. The primary aim of our study is to compare the efficacy of daptomycin versus vancomycin in the treatment of bloodstream infections due to methicillin-resistant *Staphylococcus aureus* isolates with high vancomycin minimum inhibitory concentrations (greater than or equal to 1.5 ug/ml) in terms of reducing all-cause 60-day mortality.

**Methods/Design:**

The study is designed as a multicenter prospective open label phase IIB pilot randomized controlled trial. Eligible participants will be inpatients over 21-years-old with a positive blood culture for methicillin-resistant *Staphylococcus aureus* with vancomycin minimum inhibitory concentration of greater than or equal to 1.5ug/ml. Randomization into intervention or active control arms will be performed with a 1:1 allocation ratio. We aim to recruit 50 participants over a period of two years. Participants randomized to the active control arm will receive vancomycin dose-while those randomized to the intervention arm will receive daptomycin. Participants will receive a minimum of 14 days study treatment.

The primary analysis will be conducted on the intention-to-treat principle. The Fisher’s exact test will be used to compare the 60-day mortality rate from index blood cultures (primary endpoint) between the two treatment arms, and the exact two-sided 95% confidence interval will be calculated using the Clopper and Pearson method. Primary analysis will be conducted using a two sided alpha of 0.05.

**Discussion:**

If results from this pilot study suggest that daptomycin shows significant efficacy in the treatment of bloodstream infections due to methicillin-resistant *Staphylococcus aureus* isolates with high vancomycin minimum inhibitory concentrations, we aim to proceed with a larger scale confirmatory study. This would help guide clinicians and inform practice guidelines on the optimal treatment for such infections.

**Trial registration:**

The trial is listed on clinicaltrials.gov (NCT01975662, date of registration: 29 October 2013).

## Background

Methicillin-resistant *Staphylococcus aureus* (MRSA) has emerged as one of the most common hospital-acquired pathogens worldwide. In our local setting in Singapore it is the predominant antibiotic-resistant pathogen, accounting for more than one-third of the clinical isolates of *Staphylococcus aureus* (*S. aureus*) [1]. Infection with MRSA is associated with increased morbidity, requirement of longer duration of antibiotic treatment, higher healthcare costs, prolonged hospitalization, and an increased risk of death [2-4]. This risk is higher in patients who have been suboptimally treated either with an ineffective antibiotic or inadequate surgical intervention.

In recent years, there has been an increase in the number of MRSA isolates with high vancomycin minimum inhibitory concentrations (MICs) [5]. Higher vancomycin MICs have been associated with prolonged bacteremia and increased mortality [6,7]. Vancomycin, a tricyclic glycopeptide, is the standard first-line treatment for patients with MRSA bacteremia. However, studies have linked vancomycin treatment failure in MRSA with higher vancomycin MICs even at MICs below the Clinical and Laboratory Standards Institute (CLSI) susceptibility breakpoints for *S. aureus* (≤2 ug/ml) [8-11]. Recent consensus guidelines recommend that clinicians consider using alternative agents for MRSA infection when the vancomycin MIC is greater than 1 ug/ml [12,13], especially if there is evidence of clinical failure with regards to vancomycin treatment.

Daptomycin, a lipopeptide antibiotic, is approved by the US Food and Drug Administration for the treatment of *S. aureus* bacteremia and is considered a reasonable alternative to vancomycin. The efficacy of daptomycin has been demonstrated in an open-label randomized clinical trial by Fowler *et al.*[14]. In a subgroup analysis this trial showed that the success rate for daptomycin was marginally greater (although not statistically significant) than the standard treatment among patients with MRSA bacteraemia (44% for daptomycin versus 31.8% for standard treatment; *P* = 0.28). Daptomycin, however, showed a worse outcome than standard treatment in patients with left-sided endocarditis due to *S. aureus*. A subsequent report evaluating the MRSA isolates from this study found that all strains in the vancomycin arm had an MIC of less than or equal to 1 ug/ml [15], hence it is difficult to state conclusively whether daptomycin is superior to vancomycin in the treatment of MRSA infections. Three recent cohort studies comparing daptomycin and vancomycin for bloodstream infections (BSIs) due to MRSA with a high vancomycin MIC demonstrated that daptomycin was associated with a better outcome, both in terms of rates of clinical success and mortality, compared to vancomycin [16-18]. However, being retrospective non-randomized studies, these results need to be interpreted with caution.

As there have been no head-to-head randomized clinical trials comparing the treatment efficacy of vancomycin versus daptomycin in the treatment of BSIs due to MRSA with high vancomycin MICs, the question of whether alternative treatment confers a benefit in cases of MRSA BSI with high vancomycin MIC remains. When treating such infections, clinicians are often faced with the decision of whether to continue the patient on a well-established, fairly cheap standard treatment (vancomycin), or switch to a more expensive alternative such as daptomycin from the outset.

Despite better infection control measures and awareness, we foresee that MRSA will continue to remain a problem in both our local healthcare setting and internationally over the coming years. In particular, we also expect a continued increase in the number of MRSA isolates with high vancomycin MICs. It is thus important that daptomycin is tested against vancomycin in a rigorous head-to-head randomized clinical trial to ascertain if either medication represents a more efficacious treatment strategy.

### Aims

The aim of our study is to compare the efficacy of daptomycin treatment versus vancomycin treatment in the treatment of MRSA BSIs due to isolates with high vancomycin MICs. If results from this pilot study suggest that daptomycin shows significant efficacy, we aim to proceed with a larger scale confirmatory study. This would help guide clinicians and inform practice guidelines on the optimal treatment for such infections.

### Hypothesis

The hypothesis of our trial is that daptomycin treatment is superior to vancomycin treatment in reducing the 60-day all-cause mortality rate from BSIs due to MRSA with high vancomycin MICs from 25 to 10%.

### Objectives

The primary objective is to compare the rate of all-cause mortality at 60 days post index blood culture between participants randomized to daptomycin versus those randomized to vancomycin for the treatment of MRSA BSIs due to isolates with high vancomycin MICs (≥1.5 ug/ml). Index blood culture is defined as the first blood culture which grows MRSA that has an MIC of more than or equal to 1.5 ug/ml. The key secondary objective is to compare the rates of ‘clinical failure’ between treatment arms. Other secondary objectives include comparing time to microbiological clearance, rates of nephro- and musculoskeletal toxicities, and the need to discontinue the study drug or add an additional anti-MRSA agent due to worsening infection between treatment arms.

## Methods/Design

### Study participants

In this pilot study, a minimum of 50 participants will be recruited over the course of two years, across three academic teaching hospitals in Singapore (Singapore General Hospital (SGH), National University Hospital (NUH), and Tan Tock Seng Hospital (TTSH)). Each hospital’s microbiologist will identify patients whose blood cultures are positive for MRSA with vancomycin MICs of more than or equal to 1.5 ug/ml using the Epsilometer test (E-test) method or the VITEK™-2 system (bioMerieux, Marcy l’Etoile, France) and inform the site trial coordinator. The site trial coordinator will highlight all such patients to the investigators. The primary physician will be approached by one of the investigators for verbal consent to speak to the patient about possible participation in the study. After obtaining informed consent from the patient for participation in the study, the patient will then be assessed to ensure he/she meets all the inclusion and exclusion criteria. The patient will then be randomized to either study treatment. The infectious disease physician will co-manage the patient together with the primary physician to ensure that all elements in the protocol are complied with.

During the informed consent process participants will be asked for consent regarding the archiving of residual blood specimens for future use in further elucidating pharmacokinetic and pharmacodynamic parameters as well as resistance mechanisms of MRSA. This trial has been granted ethics approval by the Singhealth Centralised Institute Review Board (CIRB) (approval ID: 2013/846/E).

All information obtained during the course of this study will be kept strictly confidential and will be kept by study site. Only the investigators and study staff are able to access the information during and after the study. The information may also be given to the health authorities, ethics committees, or other persons required by law. Identifiable information will never be used in a publication or presentation. In the event of any publication regarding this study, patients’ identity will remain confidential.

### Inclusion criteria

To be eligible for enrolment, patients must be inpatients above the age of 21 years, have MRSA bacteremia due to MRSA isolates with a vancomycin MIC ≥ 1.5 ug/ml and be prepared to undergo all study treatments and procedures and attend follow-ups as per the study protocol.

### Exclusion criteria

Subjects meeting any of the following criteria will be excluded from study participation: 1) Allergy to any of the study medications, 2) Pregnant or breastfeeding females, 3) Unable to provide consent or have no legally acceptable representatives, 4) Currently enrolled or within the past three months participated in an interventional antibiotic or vaccine trial, 5) >48 hours after MRSA vancomycin MIC ≥1.5 ug/ml confirmation by the microbiology laboratory (assessed from time of lab report), 6) On palliative care or with less than 24 hours of life expectancy (as discussed with their primary physicians), 7) Polymicrobial bacteremia, 8) Pneumonia, 9) On treatment with linezolid, tigecycline, or ceftaroline immediately prior to enrolment, 10) Previous blood cultures positive for MRSA within the preceding month, 11) On vancomycin or daptomycin treatment for more than 96 hours prior to enrolment, 12) On vancomycin or daptomycin treatment for more than 96 hours prior to enrolment, 13) Baseline serum creatine kinase (CK) more than 1.5 times the upper limit of normal, 14) Patients with prosthetic heart valves or 15) Any other significant condition that would, in the opinion of the investigator, compromise the patient’s safety or outcome in the trial.

a. Isolation of a significant organism other than MRSA from index blood cultures or blood cultures taken up to two weeks prior to enrolment and/or for which the patient is still on treatment.

b. Chest x-ray at baseline consistent with pneumonia AND at least two of the following signs and symptoms: New onset or worsening cough, purulent sputum or increased suctioning requirements, dyspnea/tachypnea or respiratory rate > 30/min, hypoxemia or worsening gas exchange as determined by the study investigator.

### Withdrawal criteria

A patient will be withdrawn from the study if the patient or legally acceptable representative withdraws consent at any time or if the investigator deems it is in the best interest of the patient due to safety concerns.

Any participant withdrawn from the study intervention will remain in follow-up for observation of safety and efficacy end-points. However, all patients are free to withdraw completely from the study at any time without giving a reason. If patients withdraw completely from study participation no further information will be collected for study purposes.

### Study design

**Figure 1:**
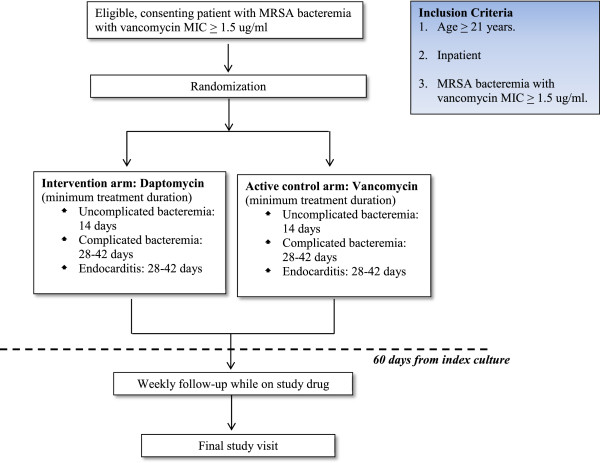
**Study schematic.** Vancomycin will be dosed intravenously at 15 mg/kg Q12h with appropriate dose adjustments by a pharmacist in patients with a creatinine clearance of less than 50 ml/min. Trough levels will be monitored pre-third or fourth dose (inclusive of doses received prior to study enrolment) and doses will be adjusted accordingly by a pharmacist to achieve a trough level of 15 to 20 ug/ml. Subsequently, vancomycin trough levels will be monitored at least every seven days (+/-three days) but additional levels may be required for dose titration at the discretion of the pharmacist or the managing physician.

This study is a multicenter prospective open-label phase IIB pilot randomized controlled trial. Participants meeting the inclusion and exclusion criteria will be randomized to one of two arms: daptomycin treatment (intervention arm) or vancomycin treatment (active control arm), as shown in Figure 1. We foresee that a large majority of the participants would have already been started on vancomycin by their primary physicians at the time of their enrolment into the trial. Enrolled participants will thus be randomized to either continuing on vancomycin or switching treatment to daptomycin. The remaining participants who have either not been started on any antibiotics or are on antibiotics other than vancomycin will be randomized similarly to either treatment with vancomycin or daptomycin.

Daptomycin will be dosed intravenously at 6 to 8 mg/kg once daily. In patients with a creatinine clearance of less than 30 ml/min or on intermittent or continuous hemodialysis daptomycin will be dosed at 6 to 8 mg/kg every 48 hours. Daptomycin will be administered after hemodialysis in patients undergoing intermittent hemodialysis. As per daptomycin approved label recommendations, statins or any other drugs associated with rhabdomyolysis will be discontinued in patients who are randomized to the daptomycin treatment arm. As there are currently no recommended guidelines for optimal daptomycin trough and peak levels, serum daptomycin peak (taken within 30 minutes of completion of daptomycin infusion) and trough levels (taken immediately before the next dosing of daptomycin) will be systematically collected on day seven (+/-three days). Aliquot samples will be archived for future pharmacokinetic and/or dynamic analysis when new data becomes available. Daptomycin trough and peak levels will only be drawn and archived in patients who consent to this.

The duration of treatment will be determined based on the type of bacteremia. Participants with uncomplicated bacteremia will receive a minimum of 14 days antibiotics and those with complicated bacteremia or infective endocarditis will receive a minimum of 28 to 42 days antibiotics from the date that microbiological clearance is achieved. Microbiological clearance is defined as two consecutive MRSA negative blood cultures. Uncomplicated bacteremia is defined as the isolation of MRSA from enrolment blood cultures in patients without endocarditis and without evidence of spread to other organs. Complicated bacteremia without endocarditis is defined as the persistence of MRSA in blood cultures beyond four days from initial positive culture, the presence of spread of infection, or infection of a vascular catheter, implantable cardiac device, or orthopedic/joint prosthetic implant not removed within four days. Right-sided endocarditis (complicated bacteremia) is defined as definite or possible endocarditis involving the tricuspid or pulmonary valve in patients without predisposing abnormalities or active infection of the mitral or aortic valves. Left-sided endocarditis (complicated bacteremia) is defined as definite or possible endocarditis involving the mitral or aortic valve. In order to make the above diagnoses, participants will be required to undergo an echocardiogram within the first 10 days of randomization. The definition of ‘definite’ or ‘possible’ endocarditis will be determined using the modified Duke criteria [19]. The diagnosis of the type of bacteremia may change during the course of study and treatment duration will be changed accordingly. This decision will be made by the managing physician.

Baseline as well as serial clinical and laboratory data will be collected as per the trial schedule (Table 1). Participants will be monitored daily by their clinical team in the hospital until discharge, with weekly assessments (with a window period of +/-three days) by the study team during the course of the study. All participants will be asked to return for a follow up assessment 60 days after the index culture (with a window period of -7 or +14 days).

**Table 1:** Study trial schedule

**Assessments**	**Pre-screening activity**	**Screening**^**2 **^**and Randomization**	**While on treatment**^**3**^																	**Follow-up period**
**Day of study**			**Pre-3**^**rd **^**or 4**^**th **^**dose of vancomycin**	**1-6**^**1**^	**7 (+/-3)**	**8-13**^**1**^	**14 (+/-3)**	**15-20**^**1**^	**21 (+/-3)**	**22-27**^**1**^	**28 (+/-3)**	**29-34**^**1**^	**35 (+/-3)**	**36-41**^**1**^	**42 (+/-3)**	**43-48**^**1**^	**49 (+/-3)**	**50-55**^**1**^	**56 (+/-3)**	**60 days after index culture**^**15 **^**(-7 or + 14 days)**
**Informed consent**		x																		
**Review of eligibility criteria**		x																		
**Randomization and dosing**^**4**^		x																		
**Demographics**		x																		
**Medical history**^**5**^		x																		
**Physical examination**^**6**^		x			x		x		x		x		x		x		x		x	x
**Adverse event monitoring**^**7**^				x	x	x	x	x	x	x	x	x	x	x	x	x	x	x	x	x
**Concomitant medication monitoring**^**8**^		x		x	x	x	x	x	x	x	x	x	x	x	x	x	x	x	x	x
**Adherence check**				x	x	x	x	x	x	x	x	x	x	x	x	x	x	x	x	
**Vancomycin MIC**^**9 **^**testing**	x																			
**FBC**^**10**^		x			x		x		x		x		x		x		x		x	
**Creatinine**^**11**^		x			x		x		x		x		x		x		x		x	
**CK**^**12**^		x			x		x		x		x		x		x		x		x	
**Vancomycin trough level**^**13**^			x		x		x		x		x		x		x		x		x	
**Daptomycin trough and peak level**^**14**^					x															
**Blood culture**	x^15^		To be done daily until two consecutive negative sets																	x
**CXR**^**16**^		x																		
**Echocardiogram**^**17**^				x																
**Urine pregnancy test**^**18**^		x																		
**Charlson comorbidity index**		x																		

^1^These assessments to be done only for patients who remain inpatients. ^2^Screening blood tests: full blood count, creatinine, creatine kinase, as well as chest radiograph will be accepted if done within 48 hours prior to screening. ^3^Duration of therapy will be determined based on type of bacteremia. ^4^Randomization and first dose of study drug may take place up to one day from screening. Eligibility criteria will be reviewed again if randomization and first dose of study drug takes place one day from screening. ^5^Full medical history including comorbidities, current medication, allergies, and presenting and current symptoms. ^6^Includes vital signs for temperature, blood pressure (systolic and diastolic), heart rate, respiratory rate, and pulse oximetry. Vital sign readings will be considered to be the readings documented by the investigator during the study visit. ^7^Adverse event monitoring will be done weekly in patients who are receiving treatment in an outpatient setting. ^8^Concomitant medication monitoring will be done weekly in patients who are receiving treatment in an outpatient setting. ^9^Mean inhibitory concentration (MIC) test results done within 48 hours prior to screening can be used. ^10^Full blood count: hemoglobin, total and differential white blood cell counts, and platelet count ^11^Patients with prior end-stage renal failure already on longterm hemodialysis or peritoneal dialysis will not require serum creatinine monitoring. ^12^Creatine kinase. ^13^For patients randomized to the vancomycin arm only. Blood for the first trough level will be drawn immediately prior to infusion of the 3^rd^ or 4^th^ dose of vancomycin (inclusive of doses received prior to study enrolment) and the dose will be adjusted accordingly to achieve a trough level of 15 to 20 ug/ml. For patients whose 3^rd^ or 4^th^ dose of vancomycin was received prior to study enrolment, a vancomycin trough level should be taken within 48 hours of enrolment. All patients will have a trough level measured at least every seven days (+/-three days) but additional levels may be required for dose titration at the discretion of the pharmacist or the managing physician. ^14^For patients randomized to the daptomycin arm only. Blood for the trough level will be drawn immediately prior to the infusion of daptomycin and blood for the peak level will be drawn 30 minutes after the infusion has completed. This will only be done once on day seven (+/-three days) of the study. ^15^The index blood culture is the first blood culture which grows MRSA that has an MIC ≥ 1.5 ug/ml obtained in the pre-screening period. ^16^Chest radiograph.^17^Echocardiogram will be done once within the first 10 days of randomization.^18^Only for female patients of child-bearing potential.

MIC = minimum inhibitory concentration, FBC = full blood count, CK = creatine kinase, CXR = chest radiograph.

All participants will be initiated on antibiotic treatment as inpatients, however, participants in either arm may receive their antibiotics at the outpatient antibiotic therapy (OPAT) clinic of the individual hospitals. Participants with end-stage renal failure on hemodialysis may receive their antibiotics at their respective hemodialysis centre. Participants will need to achieve microbiological clearance and be deemed clinically stable by their managing physicians before they can be allowed to receive their antibiotics at the OPAT clinic or the hemodialysis centre as per standard clinical practice. However, all such participants will be required to return to the site weekly for adherence checks, adverse event monitoring, and blood tests as per the trial schedule (Table 1).

### Randomization

Balanced treatment assignments at a 1:1 ratio will be achieved using permuted block randomization stratified by site, with the use of a computer-generated list of random numbers. Stratification by site is used to ensure balance between the study treatment arms across sites. Random permuted blocks are used to ensure balance over time. The block length is determined by the statistician preparing the randomization list and will not be known by the clinical investigators or site personnel. The randomization list will be generated by the Singapore Clinical Research Institute (SCRI). Randomization will be done via direct web randomization system. Authorized site personnel will randomize the patients via a password-protected internet website. The web randomization system will then determine the study treatment arm and provide a subject number to be used for the patient. Sealed opaque backup randomization envelopes will also be provided to the site in case of any internet failure.

### Study visits and procedures

Randomization will occur at the screening visit after the eligibility criteria have been met and informed consent has been obtained. The study treatment will be commenced immediately post-randomization. The screening visit will include the following procedures: written informed consent, documentation of demographic data, full medical history including comorbidities and current medications, physical examination including measurement of blood pressure, pulse rate, respiratory rate, temperature and oxygen saturation, calculation of the Charlson comorbidity index [20], baseline blood tests including full blood count (FBC), creatinine, and serum CK, calculation of creatinine clearance which will be calculated manually using the modification of diet in renal disease (MDRD) equation [21], chest x-ray (CXR), and urine pregnancy test (in females of child-bearing age). Screening blood tests and CXR will be accepted if they have been performed within 48 hours of screening. A review of the eligibility criteria will be repeated on the date of randomization and first dose of the study treatment if randomization and receipt of the first dose of the study treatment occurs one day after screening.

The following tests and assessments will be done during the course of the study: blood cultures will be performed daily until two consecutive negative sets are achieved; blood tests for FBC, creatinine and serum CK every seven days (+/-three days); vancomycin trough level pre- third or fourth dose of vancomycin for patients in the vancomycin arm (inclusive of doses received prior to enrolment) and then every seven days (+/-three days); weekly clinical assessment including a physical assessment; and an echocardiogram done at least once within the first 10 days of randomization. At day seven (+/-three days) daptomycin trough and peak levels will be taken for participants in the daptomycin arm who consent to the archiving of their blood specimens.

Participants will return 60 days (-7 or + 14 days) from time of index blood culture to determine mortality status and to obtain a blood culture. During this visit, participants will also be asked if they have had to be readmitted to hospital or have sought medical attention since discharge. The details of readmission will be sought by reviewing the participant’s medical records. In particular, we will find out if participants have had any positive blood cultures for MRSA during this period. Patients who do not achieve microbiological clearance by 60-days post index culture will be censored and will still receive treatment outside of the trial period.

If a participant does not return for the final study visit (60 days from the time of index blood culture) attempts will be made to contact the participant for up to 14 days from the date of the final study visit. If the patient has not responded by this time they will be considered lost to follow-up. Table 1 summarizes the study visits and procedures.

### Study endpoints

The primary endpoint is all-cause mortality 60 days from the date of positive index blood culture. The index culture is defined as the first blood culture which grows MRSA that has an MIC of more than or equal to 1.5 ug/ml. The date of index blood culture is the date that the patient’s blood was drawn for this blood culture.

The secondary efficacy endpoints of the study are as follows: 1) Clinical failure defined as composite of all-cause mortality 60 days from the index blood culture, microbiologic failure (defined as growth of MRSA in blood cultures more than or equal to seven days from index blood culture while the patient is still on treatment) and/or a recurrence (defined as a positive blood culture for MRSA at any point in time from the point of microbiological clearance up to 60 days from the index blood culture) of MRSA BSI, 2) Clinical failure defined as a composite of microbiologic failure and/or a recurrence of MRSA BSI, 3) Clinical failure defined a composite of all-cause mortality 60 days from the index blood culture and/or microbiologic failure and 4) Time to microbiological clearance is defined as the time from index blood culture to the first MRSA negative blood culture which is followed by a second MRSA negative blood culture

The safety endpoints include: 1) Nephrotoxicity defined as an increase in the serum creatinine level of 50 umol/L from baseline or 50% above baseline throughout the course of the study, in the absence of an alternative explanation, 2) Musculoskeletal toxicity defined by a rise in CK of five times the upper limit of normal from baseline during the course of the study, 3) The need to stop the study drug due to toxicity as defined by the Common Terminology Criteria for Adverse Events version 4.03 (CTCAE) [22], 4) The need to discontinue study treatment due to worsening infection while on study treatment.

Other exploratory endpoints include all-cause mortality 60 days from the time of the index blood culture and clinical failure of daptomycin treatment versus vancomycin treatment in the four subtypes of bacteremia (as per the patient’s final diagnosis), that is, uncomplicated bacteremia, complicated bacteremia without endocarditis, right-sided endocarditis, and left-sided endocarditis.

### Study intervention

Vancomycin and daptomycin are approved post-marketed drugs, which are routinely used in clinical practice. Each site will use the local stocks of the study drugs available at the institution at the time of dispensing. The study drugs will be stored, ordered, and dispensed as per each institution’s practice and applicable policies.

The active control arm will be vancomycin administered intravenously. Vancomycin will be dosed at 15 mg/kg every 12 hours with appropriate dose adjustments by a pharmacist in patients with a creatinine clearance less than 50 ml/min, so as to achieve a vancomycin trough level of 15 to 20 ug/ml. This dosing regimen is based on a consensus statement of the American Society of Health-System Pharmacists, the IDSA, and The Society of Infectious Diseases Pharmacists on guidelines for vancomycin dosing [12,23].

The intervention arm will be daptomycin administered intravenously. IDSA guidelines recommend a dose of 6 mg/kg every 24 hours but suggest a higher dose of 10 mg/kg every 24 hours for patients with vancomycin treatment failure and/or persistent bacteremia [13]. Some experts, however, do recommend a higher dose of at least 8 mg/kg in all cases in view of the concentration-dependent effect of the drug [24-26]. The main concern with high dose daptomycin is an increased risk of musculoskeletal toxicity, however studies have not demonstrated this. In a retrospective review of 61 patients by Fiqueroa *et al.*[24], patients received a mean dose of 8 mg/kg of daptomycin (range 7 to 11 mg/kg) for a median of 25 days. This dose of drug was well tolerated and the incidence of significant CK elevation was 4.9% which resolved after discontinuation of the drug. This rate is comparable to other studies in which a lower dose of daptomycin was used [14]. In a retrospective multicenter review of 250 adults who received daptomycin at doses more than or equal to 8 mg/kg for complicated gram-positive infections [26], only three patients (1.2%) developed an adverse event attributable to high dose daptomycin, with the event either considered mild or moderate. The median end-of-therapy CK level was within the normal range. Doses of up to 12 mg/kg have also been shown to be well tolerated in healthy volunteers [25]. In this study, daptomycin will be dosed intravenously at 6 to 8 mg/kg every 24 hours.

Patients with uncomplicated bacteremia will receive a dose of 6 mg/kg every 24 hours. Patients with suspected complicated bacteremia or endocarditis, or in receipt of at least two doses of vancomycin in the last 90 days (apart from vancomycin received for their current MRSA bacteremia) will receive a dose of 8 mg/kg every 24 hours.

If during the course of the study patients who were initially thought to have an uncomplicated bacteremia subsequently develop a complicated bacteremia or endocarditis, the dose of daptomycin will be increased from 6 to 8 mg/kg. The reverse applies to those who were initially suspected to have a complicated bacteremia or endocarditis but who subsequently were found to only have an uncomplicated bacteremia (the dose will be decreased from 8 to 6 mg/kg).

In patients with a creatinine clearance of less than 30 ml/min or on intermittent or continuous hemodialysis daptomycin will be dosed at 6 to 8 mg/kg every 48 hours. The same criteria as above applies as to whether they receive 6 or 8 mg/kg every 48 hours. Daptomycin will be administered after hemodialysis in patients undergoing intermittent hemodialysis. There are currently no recommended trough or peak level for daptomycin.

### Safety considerations

The site investigator of each study site is responsible for monitoring the safety of patients for that particular study site. Immediate medical attention should be provided to resolve any serious adverse event (SAE), which occurs during the study. All adverse events (AEs) and SAEs occurring from the time the patient has given written informed consent and during the study will be followed until resolution. AEs will be graded using the National Cancer Institute’s Common Terminology Criteria for Adverse Events (CTCAE), version 4.03 [22]. The relationship of the event to the study drug and whether the event is an expected or not will be assessed using the listing of adverse effects contained in the summary of the product characteristics for the antibiotics used. All SAEs that are unexpected and related to the study drug will be reported to the Health Sciences Authority (HSA).

An independent Data Safety and Monitoring Board (DSMB) comprising two independent physicians and a biostatistician shall be established prior to commencing the study. Interim analyses including both key efficacy and safety endpoints will be performed after the first 25 patients have been assessed 60 days from index blood culture. Any significant difference in efficacy or safety will be highlighted to the Trial Steering Committee (comprising all the site investigators and the principal investigator), along with the DSMB’s recommendations for action. If there is a significant safety concern, the DSMB may recommend to the Trial Steering Committee that the trial should be stopped.

### Data collection and storage

The quality of the data collected will be monitored regularly by the site principal investigator. Monitoring is done in order to verify that the trial is conducted in compliance with the Singapore Guideline for Good Clinical Practice.

All study staff will be trained in the execution of study procedures including data collection, and entry and training will be recorded in the study site file. The site coordinator will record data from the source documents into a central electronic database. The system will allow for audit tracking. Essential documents will be retained for a minimum of six years after completion or discontinuation of the study.

### Determination of sample size

In this pilot study we aim to evaluate the efficacy of the daptomycin versus vancomycin at 60-days post index blood culture for a decision to proceed with a larger scale confirmatory study. For this purpose, Simon’s randomized selection design is used [27], and a total of 21 participants per arm are needed so as to guarantee a 90% probability of correctly selecting the daptomycin arm as superior to the vancomycin arm if it is truly superior by a margin of 15%. This is assuming a survival rate of 75% at 60-days post index blood culture in the vancomycin arm compared to the survival rate of 90% in the daptomycin arm, based on previously published retrospective case–control and cohort studies [16-18]. This type of design is to select one of two arms as being worthy of further evaluation in a subsequent study but not to confirm the superiority of the selected arm. Assuming an attrition rate of 20%, target recruitment is a minimum 50 patients over the course of two years.

### Statistical analysis

All statistical analyses will be carried out on an intention-to-treat (ITT) basis unless otherwise stated. Continuous variables will be summarized using mean and standard deviation. Categorical variables will be summarized using the number of observations and percentages. All tests of hypotheses will be two-tailed. Statistical significance will be considered to be *P* = 0.05 unless otherwise stated. All statistical analyses will be performed using SAS Version 9.2 (SAS Institute Inc, Cary, North Carolina, USA) or higher Statistical analysis and programming support will be provided by SCRI.

An ITT population is defined as all randomized patients. The treatment group of patients in the ITT population is the planned treatment group, that is, according to the randomization list planned prior to the study commencement. A per protocol (PP) population is defined as all randomized patients who have taken at least one dose of study treatment and have had the primary endpoint measured. The treatment group of patients in the PP population is according to the treatment actually received after the randomization. Patients will be assigned to the group as per the first dose received of the study treatment after the randomization. If patients are issued the incorrect study treatment they should continue on this treatment and not switch. For example if a patient was randomized to vancomycin but the first treatment they received after randomization was daptomycin they should continue to take daptomycin. The treated population is the same as the PP population but without the requirement that the primary endpoint is measured. The treated population is defined as all randomized patients who have taken at least one dose of study treatment after randomization. The treatment group of patients in the treated population is according to the first treatment actually received after the randomization.

Demographic characteristics (age, gender, and so forth) and other baseline characteristics (such as clinical measures taken at baseline) will be summarized using descriptive statistics by treatment group for the ITT population. The primary analysis will be conducted on the ITT population. The Fisher’s exact test will be used to compare 60-day mortality from the index blood culture (primary endpoint) between the two treatment groups and the exact 95% confidence interval will be calculated using the Clopper and Pearson method. Primary analysis will be conducted using a two-sided alpha of 0.05. For the primary analysis, dropouts will be imputed as mortality. A sensitivity analysis will be performed similar to the primary analysis but using the PP population. Additionally, the time from index blood culture to death for each treatment group will be calculated using the Kaplan-Meier product-limit method for the ITT population, censoring at the 60-day post index culture assessment. Those lost to follow-up or totally withdrawn from study will be censored at the date of last follow-up. A sensitivity analysis will be performed by fitting a Cox regression model to estimate the hazard ratio and its associated 95% confidence interval. The Fisher’s exact test will also be used to compare clinical failure (according to the three definitions as described in the section ‘Study endpoints’) between the treatment arms for the ITT population and the exact 95% confidence interval will be calculated using the Clopper and Pearson method.

For the ITT population, dropouts will be imputed as ‘clinical failure’ if the patient meets the following criteria under the respective definition of the clinical failure endpoint:

Definition 1 (Moore *et al.*[16]): 1) contact could not be established or patient reported dead at the 60-day post index culture assessment; or 2) growth of MRSA in at least one blood culture seven days after index culture whilst patient is still on treatment, or if no cultures are performed after seven days of index culture and last culture (within seven days of index culture whilst still on treatment) was positive for MRSA; or 3) any positive blood culture after microbiological clearance, or in the absence of a blood culture after microbiological clearance, contact made and patient reporting any readmission to hospital.

Definition 2 (Cheng *et al.*[17]): 1) growth of MRSA in at least one blood culture seven days after index culture, or if no cultures performed after seven days of index culture and last culture (within seven days of index culture whilst still on treatment) was positive for MRSA; or 2) any positive blood culture after microbiological clearance, or in the absence of a blood culture after microbiological clearance, contact made and patient reporting any readmission to hospital.

Definition 3 (Murray *et al.*[18]): 1) contact could not be established or patient reported dead at the 60-day post index culture assessment; or 2) growth of MRSA in at least one blood culture seven days after index culture or if no cultures performed after seven days of index culture and last culture (within seven days of index culture whilst still on treatment) was positive for MRSA.

The Cox regression model will be used to compare time to microbiological clearance between the treatment groups for ITT population, censoring at the 60-day post index culture assessment. Those lost to follow-up or totally withdrawn from study will be censored at the date of last follow-up.

The analysis of safety endpoints will be performed on the treated population. The reporting period for safety data will be from the date of the first dose of study treatment to 30 days after the last dose is received. Numbers and percentages of patients calculated will be based on: 1) experiencing nephrotoxicity and musculoskeletal toxicity, 2) needing to stop the study drug due to toxicity, 3) needing to discontinue study treatment due to worsening infection, or 4) needing to have an additional anti-MRSA agent added to their treatment due to worsening infection between randomization and end of study (treatment will be reported by the study treatment arm). The frequency of AEs with severity and relation with the study treatment will be reported by the treatment arm. All SAEs will be listed by the study treatment arm and details of the events will be summarized.

## Discussion

As far as we are aware, this is the first head-to-head randomized trial comparing the safety and efficacy of daptomycin and vancomycin in the treatment of MRSA BSIs with high vancomycin MICs. If results from this pilot study suggest that daptomycin shows significant efficacy in the treatment of such infections, we aim to proceed with a larger scale confirmatory study. This would help guide clinicians and inform practice guidelines on the optimal treatment for such infections.

## Trial status

The trial has been granted a clinical trials certificate (CTC) by the Health Sciences Authority (HSA) (Certificate number: CTC1300508). Recruitment will start in February 2014.

## Abbreviations

AE: Adverse event; BSI: Bloodstream infection; CIRB: Centralised Institutional Review Board; CK: Creatine kinase; CLSI: Clinical and Laboratory Standards Institute; CT: Computed tomography; CTCAE: Common Terminology Criteria for Adverse Events; CXR: Chest X-ray; DSMB: Data Safety and Monitoring Board; FBC: Full blood count; HSA: Health Sciences Authority; IDSA: Infectious Diseases Society of America; ITT: Intention to treat; MDRD: Modification of diet in renal disease; MIC: Minimum inhibitory concentration; MRSA: Methicillin-Resistant *Staphylococcus aureus*; NUH: National University Hospital; OPAT: Out-patient Antibiotic Therapy; PICC: Peripherally Inserted Central Catheter; SAE: Serious adverse event; SCRI: Singapore Clinical Research Institute; SGH: Singapore General Hospital; TTSH: Tan Tock Seng Hospital.

## Competing interests

The authors declare that they have no competing interests.

## Authors’ contributions

SK was responsible for overall study design and drafted the manuscript. RP, MG and NNS participated in the study design and developed the statistical analysis. JGHL, SA and DL participated in the study design and will participate in the acquisition of data. TTT helped in the conception of the study, participated in the design and supervised the project. All authors read and approved the final manuscript.

## References

[B1] Hsu LY Tan TY Jureen R Koh TH Krishnan P Lin RTP Tee NWS Tambyah PA Antimicrobial drug resistance in Singapore hospitals Emerg Infect Dis 2007 13 1944 1947 18258055 10.3201/eid1312.070299 PMC2876746

[B2] Soriano A Martinez JA Mensa J Marco F Almela M Moreno-Martinez A Sanchez F Munoz I de Anta MT J Soriano E Pathogenic significance of methicillin resistance for patients with *Staphylococcus aureus* bacteremia Clin Infect Dis 2000 30 368 373 10671343 10.1086/313650

[B3] Cosgrove SE Sakoulas G Perencevich EN Schwaber MJ Karchmer AW Carmeli Y Comparison of mortality associated with methicillin-resistant and methicillin susceptible *Staphylococcus aureus* bacteremia: a meta-analysis Clin Infect Dis 2003 36 53 59 12491202 10.1086/345476

[B4] Pada SK Ding Y Hsu LY Earnest A Lee TE Yong HC Jureen R Fisher D Economic and clinical impact of nosocomial methicillin-resistant *Staphylococcus aureus* infections in Singapore: a matched case–control study J Hosp Infect 2010 78 36 40 21269733 10.1016/j.jhin.2010.10.016

[B5] Sakoulas G Mollering RC Jr Increasing antibiotic resistance among methicillin- resistant Staphylococcus aureus strains Clin Infect Dis 2008 46 S360 S367 18462091 10.1086/533592

[B6] Jacob JT DiazGranados CA High vancomycin minimum inhibitory concentration and clinical outcomes in adults with methicillin-resistant *Staphylococcus aureus* infections: meta-analysis Int J Infect Dis 2013 17 e93 e100 23089040 10.1016/j.ijid.2012.08.005 PMC3780595

[B7] van Hal SJ Lodise TP Paterson DL The clinical significance of vancomycin minimum inhibitory concentration in S*taphylococcus aureus* infections: a systematic review and meta-analysis Clin Infect Dis 2012 54 755 771 22302374 10.1093/cid/cir935

[B8] Soriano A Marco F Martinez J Pisos E Almela M Dimova VP Alamo D Ortega M Lopez J Mensa J Influence of vancomycin minimum inhibitory concentration on the treatment of methicillin-resistant staphylococcus bacteremia Clin Infect Dis 2008 46 193 200 18171250 10.1086/524667

[B9] Sakoulas G Moise-Broder PA Shentag J Forrest A Moellering RC Jr Eliopoulos GM Relationship of MIC and bacteriacidal activity to efficacy of vancomycin for treatment of methicillin-resistant *Staphylococcus aureus* bacteremia J Clin Microb 2004 42 2398 2402 10.1128/JCM.42.6.2398-2402.2004 PMC427878 15184410

[B10] Hidayat LK Hsu DI Quist R Shriner KA Wong-Beringer A High dose vancomycin therapy for methicillin-resistant *Staphylococcus aureus* infections: efficacy and toxicity Arch Intern Med 2006 166 2138 2144 17060545 10.1001/archinte.166.19.2138

[B11] Lodise TP Graves J Evans A Graffunder E Helmecke M Lomaestro BM Stellrecht K Relationship between vancomycin MIC and failure among patients with methicillin-resistant *Staphylococcus aureus* bacteremia treated with vancomycin Antimicrob Agents Chemother 2008 52 3315 3320 18591266 10.1128/AAC.00113-08 PMC2533486

[B12] Rybak M Lomaestro B Rotshcafer JC Moellering RC Jr Craig WA Billeter M Dalovisio JR Levine DP Therapeutic monitoring of vancomycin in adult-patients: a consensus review of the American society of Health-system Pharmacists, the Infectious Diseases Society of American and the Society of Infectious Disease Pharmacists Am J Heath Syst Pharm 2009 66 82 98 10.2146/ajhp080434 19106348

[B13] Liu C Bayer A Cosgrove SE Daum RS Fridkin SK Gorwitz RJ Kaplan SL Karchmer AW Levine DP Murray BE J Rybak M Talan DA Chambers HF Clinical practice guidelines by the Infectious Diseases Society of America for the management of methicillin resistant *staphylococcus aureus* infections in children and adults Clin Infect Dis 2011 52 1 38 21208910 10.1093/cid/ciq146

[B14] Fowler VG Boucher HW Corey GR Abrutyn E Karchmer AW Rupp ME Levine DP Chambers HF Tally FP Vigliani GA Cabell CH Link AS DeMeyer I Filler SG Zervos M Cook P Parsonnet J Bernstein JM Price CS Forrest GN Fätkenheuer G Gareca M Rehm SJ Brodt HR Tice A Cosgrove SE Daptomycin versus standard therapy for bacteraemia and endocarditis caused by staphylococcus aureus NEJM 2006 355 653 665 16914701 10.1056/NEJMoa053783

[B15] Rehm SJ Boucher H Levine D Campion M Eisenstein BI Vigliani GA Corey GR Abrutyn E Daptomycin versus vancomycin plus gentamicin for the treatment of bacteraemia and endocarditis due to *Staphylococcus aureus*: subset analysis of patients infected with methicillin-resistant isolates J Antimicrob Chemother 2008 62 1413 1421 18782781 10.1093/jac/dkn372 PMC2583068

[B16] Moore CL Osaki-Kiyan P Haque NZ Perry MB Donabedian S Zervos MJ Daptomycin versus vancomycin for bloodstream infections due to methicillin resistant staphylococcus aureus with a high vancomycin minimum inhibitory concentration: a case–control study Clin Infect Dis 2012 54 51 58 22109947 10.1093/cid/cir764

[B17] Cheng CW Hsu PC Yang CC Chang HJ Siu LK Wu TL Huang CT Lee MH Influence of early daptomycin therapy on treatment outcome of methicillin-resistant *Staphylococcus aureus* bacteraemia with high vancomycin minimum inhibitory concentrations Int J Antimcrob Agents 2013 41 293 10.1016/j.ijantimicag.2012.10.019 23312599

[B18] Murray KP Zhao J Davis SL Early use of daptomycin versus vancomycin for methicillin-resistant *Staphylococcus aureus* bacteremia with vancomycin MIC > 1 mg/ml: a matched cohort study Clin Infect Dis 2013 56 1562 1569 23449272 10.1093/cid/cit112

[B19] Li JS Sexton DJ Mick N Nettles R Fowler VG Jr Ryan T Bashore T Corey GR Proposed modifications to the duke criteria for the diagnosis of infective endocarditis Clin Infect Dis 2000 30 633 638 10770721 10.1086/313753

[B20] Charlson ME Pompei P Ales K MacKenzie CR A new method of classifying prognostic comorbidity in longitudinal studies: development and validation J Chronic Dis 1987 40 373 383 3558716 10.1016/0021-9681(87)90171-8

[B21] Levey AS Coresh J Greene T Stevens LA Zhang YL Hendriksen S Kusek JW Van Lente F Using standardized serum creatinine values in the modification of diet in renal disease study equation for estimating glomerular filtration rate Ann Intern Med 2006 145 247 254 16908915 10.7326/0003-4819-145-4-200608150-00004

[B22] U.S. Department of Health and Human Services Common Terminology Criteria for Adverse Events (CTCAE) Version 4.03 2010 USA: National Institutes of Health, National Cancer Institute

[B23] Rybak M Lomaestro B Rotschafer JC Moellering RC Craig WA Billeter M Dalovisio JR Levine DP Vancomycin therapeutic guidelines: a summary of consensus recommendations from the Infectious Diseases Society of America, the American Society of Health – System Pharmacists, and the Society of Infectious Diseases Pharmacists Clin Infect Dis 2009 49 325 327 19569969 10.1086/600877

[B24] Fiqueroa DA Mangini E Amodio-Groton M Vardianos B Melchert A Fana C Wehbeh W Urban CM Segal-Maurer S Safety of high-dose intravenous daptomycin treatment: three-year cumulative experience in a clinical program Clin Infect Dis 2009 49 177 180 19500039 10.1086/600039

[B25] Benvenuto M Benziger DP Yankeley S Vigliani G Pharmacokinetics and tolerability of daptomycin at doses up to 12 milligrams per kilogram of body weight once daily in healthy volunteers Antimicrob Agents Chemother 2006 50 3245 3249 17005801 10.1128/AAC.00247-06 PMC1610083

[B26] Kullar R Davis SL Levine DP Zhao JJ Crank CW Segreti J Sakoulas G Cosgrove SE Rybak MJ High dose daptomycin for treatment of complicated gram-positive infections: A large, multicentre retrospective study Pharmacother 2001 31 527 536 10.1592/phco.31.6.527 21923436

[B27] Simon R Wittes RE Ellenburg RS Randomized phase II clinical trials Cancer Treat Rep 1985 69 1375 1381 4075313

